# Compound Prunetin Induces Cell Death in Gastric Cancer Cell with Potent Anti-Proliferative Properties: In Vitro Assay, Molecular Docking, Dynamics, and ADMET Studies

**DOI:** 10.3390/biom10071086

**Published:** 2020-07-21

**Authors:** Preethi Vetrivel, Seong Min Kim, Sang Eun Ha, Hun Hwan Kim, Pritam Bhagwan Bhosale, Kalaiselvi Senthil, Gon Sup Kim

**Affiliations:** 1 Research Institute of Life science and College of Veterinary Medicine, Gyeongsang National University, Gazwa, Jinju 52828, Korea; preethivetrivel05@gmail.com (P.V.); ksm4234@naver.com (S.M.K.); sangdis2@naver.com (S.E.H.); shark159753@naver.com (H.H.K.); shelake.pritam@gmail.com (P.B.B.); 2 Department of Biochemistry, Biotechnology and Bioinformatics, Avinashilingam Institute for Home Science and Higher Education for Women, Coimbatore 641043, India; kalaiselvi_bc@avinuty.ac.in

**Keywords:** prunetin, necroptosis, gastric cancer, RIPK3, molecular docking, dynamics, ADMET

## Abstract

Gastric cancer is the common type of malignancy positioned at second in mortality rate causing burden worldwide with increasing treatment options. Prunetin (PRU) is an O-methylated flavonoid that belongs to the group of isoflavone executing beneficial activities. In the present study, we investigated the anti-proliferative and cell death effect of the compound PRU in AGS gastric cancer cell line. The in vitro cytotoxic potential of PRU was evaluated and significant proliferation was observed. We identified that the mechanism of cell death was due to necroptosis through double staining and was confirmed by co-treatment with inhibitor necrostatin (Nec-1). We further elucidated the mechanism of action of necroptosis via receptor interacting protein kinase 3 (RIPK3) protein expression and it has been attributed by ROS generation through JNK activation. Furthermore, through computational analysis by molecular docking and dynamics simulation, the efficiency of compound prunetin against RIPK3 binding was validated. In addition, we also briefed the pharmacokinetic properties of the compound by in silico ADMET analysis.

## 1. Introduction

Gastric cancer (GC) is a kind of common malignancy type that occurs in the gastrointestinal tract with the burden of second mortality rate around the world. With earlier stages being asymptomatic, by the time of diagnosis it reaches the advanced stages of malignancy [1]. Presently, the commonly employed methods like surgical resection and chemotherapeutics are still far from satisfactory outcomes, which makes it largely dismal [2]. The use of chemotherapeutics to treat various cancers at clinical trials leads to failure with increased limitations in terms of high toxicity. Accordingly, there is an urge to develop a new class of potentially safe drugs with effective action and reduced rate of cytotoxicity on normal cells [3]. The focus of attention in the prevailing days is on the use of natural compounds with anti-inflammatory, anti-allergic, antimicrobial, anti-oxidant, and anti-tumor activities as candidate drugs [4].

Programmed cell death is a common form of targeting tumor cells through apoptosis which provide multiple potential targets and strategies for developing anti-cancer drugs. Most of the current anti-cancer drugs kill the cancer cells by triggering apoptosis and its related pathways [5]. Alternative approaches that can induce cancer cell death via nonapoptotic process may provide new insights in solving problems like development of apoptotic resistance and sensitivity of tumor cells against drugs [6]. Necroptosis is an important form of programmed cell death mechanism, which is independent of apoptosis and does not involve the activation of the caspase family of proteins [7]. Necroptosis is defined relatively as a regulated form of necrotic cell death which is characterized by the morphological pattern of necrosis, involving the swelling of organelles, membrane rupture, and degradation of cells [8]. The invention of necroptosis mediated cell death will lead to the development of a novel therapeutic basis for alternative tumor treatment other than inducing apoptotic cell death [9].

Necroptosis is mediated by the activation of receptor-interacting kinase-3 (RIPK3) with mixed lineage kinase like (MLKL) protein [10]. RIPK3 belongs to the class of serine/threonine protein kinase family that promotes necroptosis induced by receptor-interacting kinase-1 (RIPK1), and MLKL is the main substrate for RIPK3 kinase activity to target the plasma membrane in inducing necroptosis cell death [11]. The expression of RIPK3 is been varied in different cell types, making it crucial for undergoing necroptosis and other pathways. Studies have also highlighted the additional role of RIPK3 in promoting other signaling pathways such as mitogen activated protein kinase (MAPK), and nuclear factor kB (NF-kB)-dependent transcriptional responses that eventually lead to necroptotic cell death [12]. Thus, targeting RIPK3 through necroptosis mediated cell death would be a promising approach in treating cancer cells.

The effectiveness and recent advances in the field of in silico analysis tools to characterize the molecular interactions serves as an important device for drug discovery [13]. Molecular docking is one of the widely adapted approaches applied to visualize the interaction among the protein and small molecule ligand at the atomic level [14]. Similarly, molecular dynamics (MD) simulations-based free energy computational methods are used to impart knowledge on the drug and protein binding pocket in a simulated environment, determining the free energy convergence when it freely moves [15]. Thus, virtual drug screening based on molecular docking technology and simulation models has become a widely accepted strategy for effective drug development [16]. 

The development of in silico models can also represent the physicochemical parameters, ADME properties, and toxicity evaluation of the drug molecules. The emphasis of a modeling approach with drug-likeness prediction provides potential merits in the application of drug discovery [17]. With the use of structure-based screening of drug molecules by ADMET prediction tools, the properties of candidates can be evaluated in terms of human intestinal absorption (HIA), blood–brain barrier (BBB) penetration, inhibition of cytochrome P450 2D6, plasma protein binding, aqueous solubility, and toxicity [18]. This aids in the exclusion of lead compounds that have low drug ability which reduces the cost and improves the efficacy of the drug in the development of pharmaceuticals [19]. 

Prunetin (PRU) is one representative O-methylated flavonoid from the group of isoflavone extracted from several source of plants [20]. The compound has been shown to exhibit numerous beneficial activities such as anti-inflammatory, anti-obesity, stress response, and also regulating proteolytic activity [21]. Prunetin has been found to possess anti-obesity potential and has involved in the inhibition of aldehyde dehydrogenase enzyme in human liver [22,23]. Prunetin is also shown to regulate the proteolytic activity in articular chondrocytes in conditions of osteoarthritis [24]. However, to the best of our knowledge there is no reported data on the anti-tumor potential of PRU on human cancer cells. In the current study, we investigated the anti-proliferative effect of the compound PRU extensively with in vitro and in silico approaches. We elucidated the in vitro cytotoxic potential and mechanism of cell death initiated by PRU on AGS, human gastric cancer cell line. Furthermore, we performed in silico analysis by molecular docking with the necroptosis target protein RIPK3 and molecular dynamics simulations were conducted to elucidate the stability and conformational changes of the PRU-RIPK3 complex. Additionally, the pharmokinetic properties of the compound in terms of drug likeness were also predicted and extensively reported.

## 2. Materials and Methods 

### 2.1. Cell Culture and Reagents

Human gastric cancer cell line AGS and human keratinocyte HaCaT cells was obtained from the Korean Cell Line Bank (Seoul, Korea). The cells were maintained in RPMI 1640 and DMEM medium supplemented with 10% heat inactivated FBS from GIBCO (Thermo Fisher Scientific, Inc, Waltham, MA, USA), and 100 U/mL penicillin, and 100 μg/mL streptomycin at a humidified atmosphere of 95% air and 5% CO_2_ in an incubator. The compound prunetin was purchased from Sigma-Aldrich Co Ltd. (St. Louis, MI, USA).

### 2.2. Measurement of Cytotoxicity of the Compound

Cell viability was determined using MTT assay. AGS gastric cancer cells and normal keratinocyte HaCaT cells were seeded at a density of 1 × 10^4^ cells/well in a 96 well plate and allowed to grow for 24 h at 37 °C in a 5% CO_2_ incubator, the cells were treated with different concentrations of prunetin at 0, 10, 20, 40, 80, and 100 μM respectively. MTT assay was carried out after 24 h of incubation. 100 mL of 0.5% (w/v) MTT dissolved in 1× PBS was added to each well and incubated for 3 h at 37 °C in dark condition. The medium was aspirated and the formazan crystals contained in the cell were solubilized by 100 μL of DMSO. After 15 min of shaking, the absorbance was read at 540 nm with a microplate reader. Similarly, MTT assay was carried out at different time interval (24 h, 48 h, and 72 h) following the above procedure. The experiments were repeated up to three times and the average of the independent experiments were considered for statistical analysis.

### 2.3. DNA Fragmentation Assay

AGS cells were seeded at a density of 4 × 10^5^ cells on 100 mm plates for the DNA fragmentation assay and incubated at 37 °C in a 5% CO_2_ incubator for 24 h. The cells were treated after overnight growth with specified PRU concentrations (0, 40, and 80 μM). The cells were harvested after 24 h treatment, and the amount of total DNA was extracted by performing lysis using lysis buffer containing 1% NP-40 in 20 mM ethylenediaminetetraacetic acid (EDTA), 50 mM Tris-HCl, and pH 7.5 for 30 min. The cell lysate was centrifuged at 3000 rpm for 5 min and the supernatant was gathered. The collected supernatant was incubated for 2 h at 56 °C in a water bath with 10 μL of 10% sodium dodecyl sulfate (SDS) solution and 5 μL of 100 mg/mL RNase A. Followed by protein digestion was performed by adding 10 μL of 25 mg/mL proteinase K enzyme and incubated at 37 °C for 2 h, then about 65 μL of 5 M NaCl was added and the contents were mixed thoroughly with 500 μL of ice-cold ethanol. The mixture was subjected to incubation at −80 °C for 2 h. It was then centrifuged for at 12,000 rpm 20 min and then the pellet was washed with 1 mL of 80% ice-cold ethanol and air-dried for 10 min at room temperature. The pellet was dissolved using 20 μL of Tris-EDTA (TE) buffer. The total DNA sample was then subjected to electrophoresis of 1.5% agarose gel and the UV light absorbance of the DNA bands were visualized.

### 2.4. Observation of Cell Death by Hematoxylin Staining

Cell death was analyzed by performing hematoxylin staining assay. AGS cells were plated on 6-well plates at a density of 1 × 10^5^ cells. After overnight growth at 37 °C in a 5% CO_2_ incubator, the cells were treated with indicated concentrations of prunetin (0, 20, 40, and 80 μM). Upon treatment for 24 h, the cells were washed with 1× PBS for two to three times and then fixed with 4× formaldehyde solution for 1–2 h at room temperature. After fixation, the cells were further washed with 1× PBS for about two times and then 500 μL per well of Mayer’s stain (Cancer Diagnostics, Inc., Durham, NC, USA) was added. The cells were stained by keeping it for about 20–30 min at room temperature. The stained cells were then viewed under microscope and photographs were taken upon using 90% glycerol as mounting solution.

### 2.5. Analysis of Cell Cycle Distribution by Flow Cytometry

The distribution of cell cycle on AGS cells treated with PRU was measured using flow cytometry analysis. AGS cells were seeded at a density of 5 × 10^5^ cells/well in a 60-mm plate and treated with indicated concentrations of PRU (0, 20, 40, and 80 μM) for 24 h at 37 °C. The cells were washed with ice-cold PBS after incubation, then trypsinized and centrifuged (1000× *g*) for 5 min. The pellet containing the cells were fixed in 70% ethanol for about 30 min at 4 °C. After fixation, the cells were subjected to washing with PBS, followed by staining with propidium iodide (50 μg/mL) including RNaseA (0.1 mg/mL). The sample was kept for 30 min in dark prior to analysis using flow cytometer. Flow cytometry analyses were performed with Cytomics FC 500 (Beckman Coulter, Brea, CA, USA). In each sample, 10,000 cells were sorted approximately. The data obtained were analyzed by using CXP Software (Beckman Coulter, Inc., Fullerton, CA, USA).

### 2.6. Investigating the Mechanism of Cell Death by Annexin V-Propidium Iodide Staining

The population of cell death on AGS cells treated with PRU was measured detected using allophycocyanin (APC)/annexin V apoptosis detection kit according to the manufacturer’s protocol (BD Biosciences, San Diego, CA, USA). AGS cells were seeded at a density of 5 × 10^5^ cells/well in a 60 mm plate and treated with indicated concentrations of PRU (0, 20, 40, and 80 μM) for 24 h at 37 °C. After incubation, the cells were harvested, washed with PBS, and then re-suspended in binding buffer that comes along with the kit. Furthermore, staining was performed by adding APC/annexin V and propidium iodide (PI) to the cells by keeping at room temperature in dark for about 30 min without adding further binding buffer. Flow cytometry analysis was performed on the cell suspensions and the data obtained were analyzed using a fluorescence-activated cell sorting machine (FACSVerseTM flow cytometer; BD Biosciences, Franklin Lakes, NJ, USA). In total, 10,000 events per sample were sorted and the data were analyzed using BD FACSuiteTM software (BD Biosciences, Becton & Dickson, Mountain View, CA, USA).

### 2.7. Reactive Oxygen Species Determination

The measurement of ROS generation on PRU treated AGS cells were performed using reactive oxygen species detection reagents kit by molecular probes, supplied by Invitrogen detention technologies. AGS gastric cancer cells were seeded at a density of 1 × 10^4^ cells/well in a 96 well plate and allowed to grow for 24 h at 37 °C in a 5% CO_2_ incubator, the cells were treated with indicated concentrations of prunetin 0, 20, 40, and 80 μM respectively. Similarly, other group co-treatment with JNK inhibitor SP100125 at 10 μM then followed by PRU treatment (0, 20, 40, and 80 μM) respectively. Molecular probe derivative of fluorescein carboxy-H_2_DCFDA (C4000) was prepared from stock solution by dissolving in 100% ethanol freshly before experiment. About 5 µL of the working solution of the dye was added to the treated AGS cells and the mixture was incubated not later than 15 min. The intensity developed was measured in fluorescent absorbance spectrometer with an excitation/emission range of 492/520 nm and the relative amount of ROS generated was quantified.

### 2.8. Analysis of Protein Expression by Western Blot

The expression of proteins on AGS cells treated with PRU was measured using western blot analysis. AGS cells were seeded at a density of 5 × 10^5^ cells/well in a 60-mm plate and treated with indicated concentrations of PRU (0, 20, 40, and 80 μM) for 24 h at 37 °C. After incubation, the cells were harvested and the protein content was lysed using radioimmuno-precipitation assay (RIPA) buffer (iNtRON Biotechnology, Seoul, Korea) containing phosphatase and protease inhibitor cocktail (Thermo Scientific, Rockford, IL, USA) by keeping it in ice for 30 min. Furthermore, the protein lysates were centrifuged at 10,000 rpm for 10 min at 4 °C and the extracted protein concentrations were determined using a Pierce™ BCA assay (Thermo Fisher Scientific, Rockford, IL, USA). Protein samples were prepared by mixing the required concentration amount with 5× sample buffer and allowed to be kept at 100 °C for 5 min. Electrophoresis was carried out on 8–15% SDS polyacrylamide gels and then allowed to separate based on the molecular weight of each proteins. The separated proteins were transferred onto a polyvinylidene difluoride (PVDF) membrane by electrophoretic mode and the membranes were blocked with 5% BSA (bovine serum albumin) or 5% Phosphoblocking solution. After blocking for 1 h at room temperature, each protein separated with specific molecular weight range was incubated at 4 °C with β-Actin (1:10,000), PARP (1:1000), Cl.PARP (1:1000), RIPK3 (1:1000), p-RIPK3 (1:1000), MLKL(1:1000), p-MKL (1:1000), JNK (1:1000), p-JNK (1:1000), p38 (1:1000), p-p38 (1:1000), ERK (1:1000), and p-ERK (1:1000) primary antibodies overnight. The incubated membranes were washed with TBS-T buffer at 10 min interval for at least five minutes and then subjected to secondary antibody incubation using horseradish peroxidase (HRP)-conjugated for 3 h in 1:1000 dilution at room temperature. The obtained protein blots were developed under an electrochemiluminescence (ECL) detection system (Bio-Rad Laboratory, Hercules, CA, USA). Protein quantification was analyzed using ImageJ software program (U.S. National Institutes of Health, Bethesda, MD, USA). The densitometry readings of the protein bands were normalized by comparing them with the expression of β-Actin.

### 2.9. Molecular Docking and Dynamics Simulation Studies

For the execution of molecular docking, the structure protein target was obtained through homology modeling using Swiss-model program from (https://swissmodel.expasy.org/). The three-dimensional structure of the compound prunetin and reference compound Ponatinib was obtained from PubChem (https://pubchem.ncbi.nlm.nih.gov/). The protein and ligand were subjected to docking using USCF Chimera software (https://www.cgl.ucsf.edu/chimera/) and all the possible conformations were obtained with default parameters. The results were evaluated based on the estimated free energy of binding and total intermolecular energy. The docked complexes were allowed to undergo molecular dynamics simulations using Desmond by D.E.Shaw Research [25,26] as discussed by Gade et al., 2018. The binding mode was subjected to simulation using TIP4PEW water solvent model and neutralized by adding counter ions. During this process, the protein backbone was restrained, and the periodic boundary conditions were fostered to avoid bad effects. Thereafter, the MD run was conducted for 10 ns and the results were visually analyzed. 

### 2.10. In Silico ADMET Prediction

The pharmacokinetics and drug-likeness prediction of the compound prunetin was evaluated by online tool SwissADME41 of Swiss Institute of Bioinformatics (http://www.swissadme.ch/) and the individual ADME behaviors of the compound was predicted. The 2D structural model of the compound was obtained from PubChem and SMILES of the compound was translated using the SMILES generator found in SwissADME and the analysis was carried out to check the various ADMET properties in terms of pharmacokinetics and drug-likeness.

### 2.11. Statistical Analysis 

All the experimental results were expressed as the mean ± standard error of the mean (SEM) of triplicate samples. Significant differences between groups were calculated by Student two-sample *t*-test assuming equal variances and *p* value < 0.05 was considered statistically significant. 

## 3. Results and Discussion

**Figure 1:**
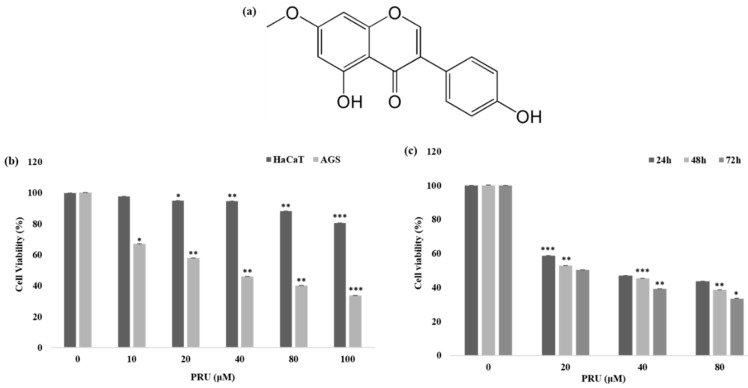
Cytotoxic properties of the compound prunetin (PRU). (**a**) Chemical structure of the compound PRU. (**b**) Plot of cell viability measured by MTT assay on AGS cancer cells and human keratinocytes, and HaCaT cells with various concentrations of PRU (0, 10, 20, 40, 80, and 100 μM) respectively. Cell viability is represented in percentage relative absorbance compared to the controls. (**c**) Plot of cell viability measured by MTT assay on AGS cancer cells at three indicated concentrations (20, 40, and 80 μM) at three different time intervals from 24, 48, and 72 h respectively. Values are given as the mean ± standard error of the mean (SEM) of three independent experiments. * *p* < 0.05 vs. control, ** *p* < 0.01 vs. control, *** *p* < 0.001 vs. control.

The cytotoxicity of the compound prunetin (PRU) was determined in AGS, gastric cancer cell, and normal human keratinocytes (HaCaT cells). AGS and HaCaT cells were treated with indicated concentrations 0, 10, 20, 40, 80, and 100 μM of PRU, respectively. The cell viability was determined 24 h later using 3-(4,5-dimethylthiazol-2-yl)-2,5-diphenyltetrazolium bromide (MTT) assay. The results shown in Figure 1b reveals that treatment with PRU inhibited the growth of AGS cells whereas comparatively, PRU treatment did not affect the cell growth of HaCaT cells. These results indicate that PRU inhibited cell growth specifically on gastric cancer cells without affecting the normal cells. Furthermore, the inhibition of cell growth on AGS cells at different time intervals—24, 48, and 72 h—was measured in three fixed concentrations (20, 40, and 80 μM) which also showed significant cell growth inhibition as represented in Figure 1c. With these cytotoxicity results, it suggests that PRU inhibits cell growth in AGS cancer cells in both dose- and time-dependent manner. Also, the cytotoxic inhibition of AGS cancer cell growth by PRU is found to be effective at a lower concentration level within 100 μM compared to the reported flavonoid monomers like pectolinarigenin and scutellarein [27,28]. From this cytotoxicity assessment, further experiments were carried out in the three indicated concentrations (20, 40, and 80 μM) of PRU, respectively.

**Figure 2:**
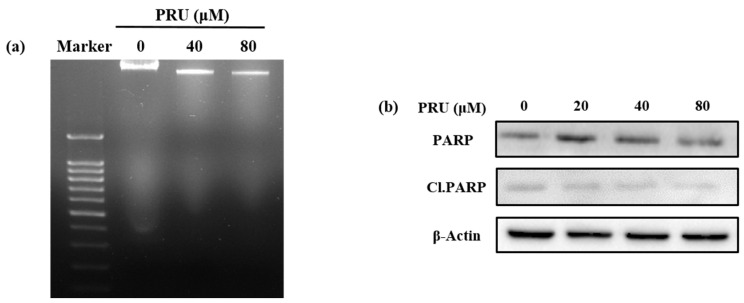
Analysis of apoptosis induction by prunetin. (**a**) Nuclear fragmentation assay on AGS cells treated with two concentrations of PRU (40 and 80 μM), respectively. (**b**) Western blot analysis of important apoptotic marker proteins PARP and Cl.PARP on PRU (20, 40, and 80 μM) treated AGS cells for 24 h.

Upon identification of cell growth inhibtion by PRU, investigations were undertaken to check whether the cell death caused by PRU was mediated by apoptosis. To determine the involvement of apoptosis, morphological observations using phase contrast microscopy, DNA fragmentation assay, and western blot analysis were performed. The results of morphological pattern observed were found to show no apoptotic features like formation of apoptotic bodies; instead, the cells exhibited loss of cell membrane integrity and star shaped cell morphology was observed which is provided in Figure S1. Also, the results of DNA fragmentation assay do not show any visible fragmented nuclei in AGS cells treated with two concentrations of PRU (40 and 80 µM), which is an important indicator of non-apoptotic cell death, shown in Figure 2a. The mechanism of apoptosis can be triggered by many factors and signals, among which the activated caspases are important effectors. The caspase activation leads to the cleavage of proteins including lamins, DNA-dependent protein kinase (DNA-PK), and poly ADP ribose polymerase (PARP) [29]. The protein poly ADP ribose polymerase (PARP) is reported as an important nuclear enzyme cleaved during DNA injury that imparts cellular dysfunction and cell death through apoptosis [30]. Western blot analysis of the apoptotic marker protein poly ADP ribose polymerase (PARP) and its cleaved form (Cl.PARP) showed no significant increased expression in AGS cells upon treatment with PRU as shown in Figure 2b. These results suggest that the cell death caused by PRU was not induced or associated with apoptosis.

**Figure 3:**
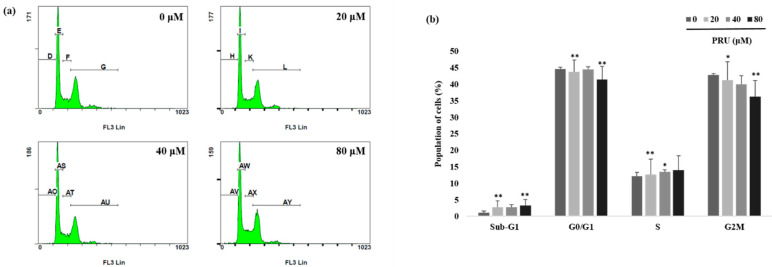
Analysis of cell cycle by flow cytometry. (**a**) The effect of PRU on cell cycle arrest was examined by flow cytometry analysis, AGS cells were treated with PRU at indicated concentrations (20, 40, and 80 μM) for 24 h. Followed by propidium iodide (PI) staining and subjected to flow cytometer analysis. (**b**) The population of cells identified on different phases of the cell cycle were represented graphically. Values are given as the mean ± standard error of the mean (SEM) of three independent experiments. * *p* < 0.05 vs. control, ** *p* < 0.01 vs. control, *** *p* < 0.001 vs. control.

The event of cell growth and cell survival process are regulated by pathways that involves various cell cycle checkpoints in relation with programmed cell death and DNA repair mechanism [31].With observed induction of cell death by microscopic examinations and cytotoxicity assay, further investigations on the mechanism of cell cycle arrest by PRU on AGS cells was carried out using flow cytometry analysis. The results obtained as shown in Figure 3a,b reveal that there is no cell cycle arrest caused by PRU in any of the phases. From this, it was found that PRU induces cell death, but does not cause any cell cycle arrest on AGS cells. 

**Figure 4:**
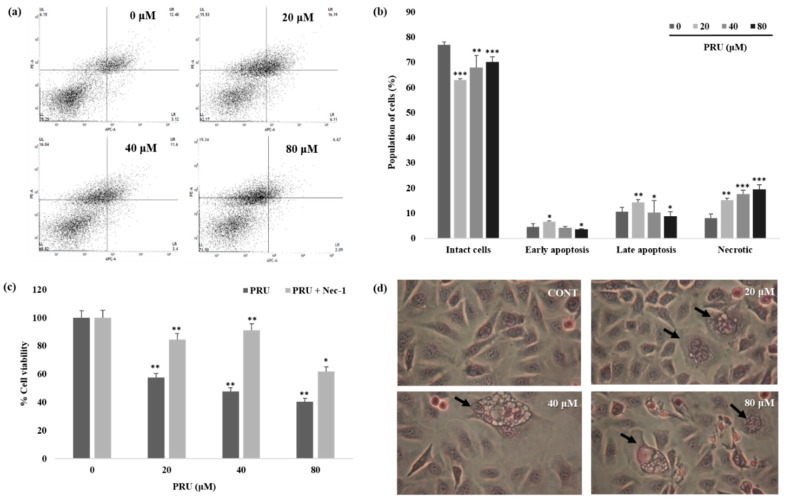
Effect of prunetin on necroptosis related cell death. (**a**) The effect of PRU on cell cycle arrest was examined by flow cytometry analysis, AGS cells were treated with PRU at indicated concentrations (20, 40, and 80 μM) for 24 h. Followed by allophycocyanin (APC)/annexin V and propidium iodide (PI) double-staining was performed which was analyzed by flow cytometry. (**b**) The population of cells identified on the different state of cell death were represented graphically. (**c**) Cell viability assay performed on PRU treated AGS cells with and without co-treatment with necrostatin-1 inhibitor (0.1 mM). (**d**) Hematoxylin staining on PRU treated AGS cells with visible necroptic morphology such as cell swelling, visible disruption of cell organelles, and formation of vacuoles. Values are given as the mean ± standard error of the mean (SEM) of three independent experiments. * *p* < 0.05 vs. control, ** *p* < 0.01 vs. control, *** *p* < 0.001 vs. control.

Furthermore, to explore the exact mechanism of cell death, allophycocyanin (APC)/annexin V and propidium iodide (PI) double-staining by flow cytometry was performed on PRU treated AGS cells. The results obtained reveal that there is a substantial increase in the proportion of cells in necrotic states when compared to the apoptotic fraction of cell population which is shown in Figure 4a,b. The data obtained through double staining using APC/PI suggest that PRU induces necrotic related cell death in AGS cells. 

Necrostatin-1 (Nec-1) is a specific small molecule potent inhibitor of necroptosis and it can specifically inhibit RIP1-RIP3 interaction [32,33]. Thus, to confirm the involvement of necroptic cell death, the cell viability of AGS cells were measured upon co-treatment with necroptosis inhibitor Nec-1 (0.1 mM) followed by PRU treatment (20, 40, and 80 μM). From the results obtained by assessing the cytotoxicity levels by MTT, it was observed that the growth of AGS cells inhibited by PRU treatment were reversed upon co-treatment with Nec-1 as shown in Figure 4c. Additionally, hematoxylin staining on PRU treated AGS cells showed visible necrotic cell death morphology including swelling of organelles, loss of plasma membrane integrity, and formation of vacuoles which are the primary characteristic features of necroptosis [34] as shown in Figure 4d. Collectively, these data suggest that compound PRU may induce necroptotic cell death in AGS gastric cancer cells.

**Figure 5:**
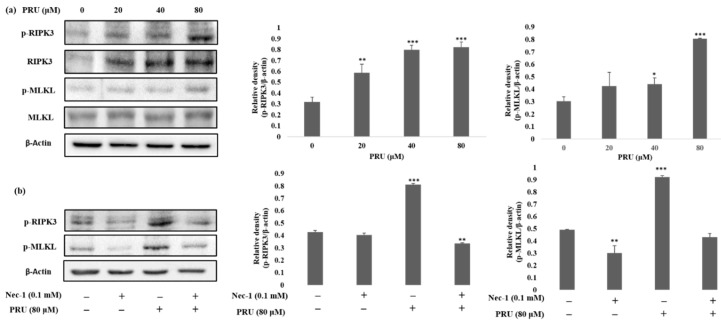
PRU induces necroptosis related cell death via RIPK3 and MLKL expression in AGS cells. (**a**) Western blot analysis of necroptosis protein markers RIPK3, p-RIPK3, MLKL, and p-MLKL protein expression on PRU treated AGS cells. The expression levels of phosphorylated form of RIPK3 and MLKL are represented graphically based on its densitometry. (**b**) Protein expression of p-RIPK3 and p-MLKL analyzed by western blot on PRU treated AGS cells with and without co-treatment with necrostatin-1 inhibitor (0.1 mM). The expression of the protein levels is represented graphically based on its densitometry. Values are given as the mean ± standard error of the mean (SEM) of three independent experiments. * *p* < 0.05 vs. control, ** *p* < 0.01 vs. control, *** *p* < 0.001 vs. control.

The event of necroptosis mediated cell death is as caspase-independent regulated type of cell death that involves the formation of necrosome complex which is mainly composed of the receptor-interacting protein kinase-1 (RIPK1), receptor-interacting protein kinase-3 (RIPK3), and mixed lineage kinase domain-like protein (MLKL) respectively [35]. With the obtained results from morphological observations and double staining supporting the induction of necroptosis by PRU, and the necroptosis marker proteins RIPK3, MLKL, the phosphorylation of RIPK3 and MLKL was evaluated by western blot analysis. The results obtained from western blot show that there is an increase in the expression of RIPK3 and MLKL along with its phosphorylated forms p-RIPK3 and p-MLKL elevated in PRU treated cells as shown in Figure 5a. 

Necroptosis signaling converges in the assembly of cytosolic component necrosome formed by the activation of effector molecule MLKL [36]. RIPK3 is a key component involved in the formation of necrosome-driven activation by phosphorylation of MLKL, which is the chief executioner of necroptosis [37]. The over expression or elevated levels of RIPK3 protein has shown to induce cell death and activation of inflammatory mediated mechanism in cancer cell lines [38]. Additionally, to further confirm whether the induction of necroptosis has led to the elevated expression of p-RIPK3 and p-MLKL, inhibitor assay was performed. Western blot was performed on the proteins p-RIPK3 and p-MLKL in PRU-treated cells upon co-treatment with inhibitor Nec-1 at 0.1 mM concentration. Nec-1 is a small effective molecule that inhibits the process of necroptosis by causing reduction in RIPK1/RIPK3 kinase activity leading to the inhibition of several residues of serine-phosphorylation during the formation of the necrosome complex [35,39]. The results as depicted in Figure 5b show that the elevated expression of p-RIPK3 and p-MLKL in the PRU treated cells were found to be reduced upon co-treatment with the inhibitor Nec-1. This supports that the inhibitor has a positive effect on the action of necroptosis cell death induced by PRU in AGS cells. Taken together, these data reveal that PRU activated RIPK3 expression leads to the phosphorylation of MLKL, inducing necroptosis cell death. 

**Figure 6:**
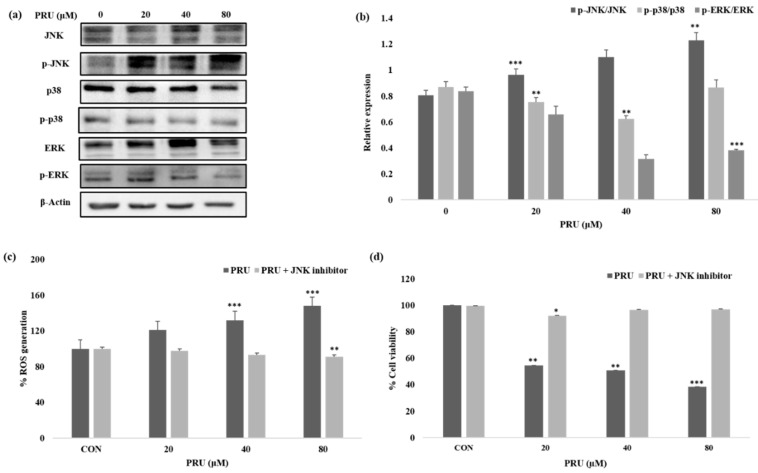
PRU induces cell death by ROS generation through JNK activation. (**a**) Western blot analysis of JNK, p-JNK, p38, p-p38, ERK, and p-ERK on PRU treated AGS cells. (**b**) The expression of phosphorylated proteins p-JNK, p-p38, and p-ERK are represented graphically based on their densitometry. (**c**) The effect of ROS generation was measured by fluorescent intensity detection in AGS cells with and without co-treatment with specific JNK inhibitor SP100125 (10 μM). (**d**) Cell viability assay performed on PRU treated AGS cells with and without co-treatment with JNK inhibitor SP100125 (10 µM). Values are given as the mean ± standard error of the mean (SEM). * *p* < 0.05 vs. control, ** *p* < 0.01 vs. control, *** *p* < 0.001 vs. control.

Given the impressive results observed in AGS cell line by PRU treatment, we were curious to determine the mechanism of its action or pathway leading to necroptosis cell death. The mitogen-activated protein kinase (MAPK) pathway is an important signaling pathway involved in oxidative stress, necroptosis, inflammation, and various pathological mechanisms [40]. In this aspect, we studied the expression levels of mitogen-activated protein kinase (MAPK) family of proteins JNK, p38, ERK, and its phosphorylated active forms—p-JNK, p-p38, and p-ERK—by western blot analysis. As shown in Figure 6a, among the MAPK family of proteins, the protein JNK has been shown to be activated with visible increase in its phosphorylated form p-JNK in a dose-dependent manner. Whereas, comparatively, the phosphorylated forms of other proteins p-p38 and p-ERK does not show significant increase in its expression level upon treatment with PRU in AGS cells as shown in Figure 6b. Sustained increase in JNK activation has been proposed in causing cell death via cytochrome c release in mitochondria [41]. Also, treatment of cancer cells has been reported to trigger necroptosis through RIPK3 complex with JNK activation and increased mitochondrial ROS levels [42].

ROS are important highly reactive inducers of cellular malfunctions that can damage DNA, proteins, and lipids. Thus ROS inducers are employed in treatment strategies in clinical trials preferentially for killing cancer cells [43]. Necrosomes have been reported to impair mitochondrial energy metabolism by disturbing ROS homeostasis, leading to cell death [42]. A growing number of reports also suggest that ROSs could disturb the mitochondrial disturbance in the event of inducing necroptosis cell death [44,45]. To uncover the relation of ROS generation through JNK activation, the amount of intracellular singlet oxygen generated in AGS cells treated with PRU (0, 20, 40, and 80 μM) was measured with or without specific JNK inhibitor (SP100125) at 10 μM concentration. The accumulation of ROS was measured upon 24 h incubation through spectroscopic fluorescence. Our results as presented in Figure 6b, showed an increased level of ROS in the group of cells treated with only PRU compared to the control, whereas the group of cells co-treated with JNK inhibitor did not show steady increase in the generation of ROS. These results show the significant correlation between the activation of JNK and ROS generation in PRU treated AGS cells. Thus, it suggests that PRU initiated the activation of JNK, leading to ROS accumulation and subsequently causing necroptosis cell death. 

Similarly, to establish the correlation of cell growth inhibition via JNK activation, the percentage of cell viability upon co-treatment with JNK inhibitor on PRU treated AGS cells were analyzed using MTT assay. Results indicate that the growth of AGS cells were inhibited in the only PRU treated group, whereas the cell viability was found to not be affected in the co-treatment group with JNK inhibitor as shown in Figure 6b. This data further supports that JNK has contributed to the induction of necroptosis cell death in PRU treated AGS cells. 

**Figure 7:**
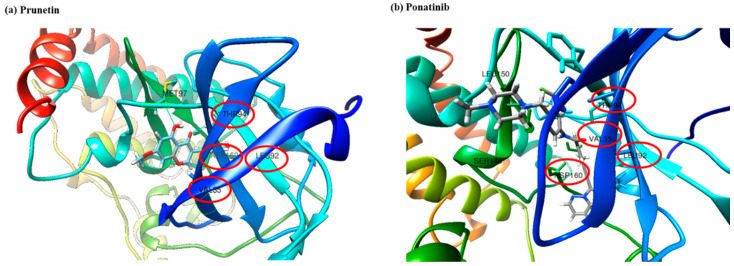
In silico molecular docking analysis of the ligands prunetin and ponatinib at the same binding pockets of the target. (**a**) The 3D structure of RIPK3 bound efficiently with compound prunetin with its interacting amino acids MET97, THR94, LEU92, VAL35, and ASP160. (**b**) The 3D structure of RIPK3 bound efficiently with the known inhibitor compound ponatinib with its interacting amino acids LEU150, ASP160, VAL35, SER146, THR94, and LEU92. The common amino acid residues involved in the interaction are circled in red color.

Encouraged with these in vitro results, the next step was to determine if the efficacy would also be observed in silico. Investigation on the interaction of RIPK3 and PRU was performed through molecular docking analysis. Structure of the compound RIPK3 was modelled by homology modeling using Swiss-Model program (https://swissmodel.expasy.org/). Furthermore, the three-dimensional structure of RIPK3 was made to undergo ligand docking with the structure of PRU and a known inhibitor of RIPK3 (ponatinib) adopted as a reference compound. To the best of our knowledge, there is no well-established agonist compound for the activation of RIPK3 protein. Thus, molecular docking approach was studied by comparing with a reported inhibitor (ponatinib), which is an allosteric inhibitor that can bind on the allosteric site of the protein RIPK3 to produce its mechanism of action [46]. Molecular docking results show that the two ligands (prunetin and ponatinib) have occupied the same binding pocket of the target RIPK3 as shown in Figure 7a,b. The molecular dock score has been put forth that the reference compound ponatinib as revealed an estimated free binding energy of −8.9 kcal/mol and the compound PRU generated an estimated free binding energy of −8.6 kcal/mol. Interestingly, both prunetin and ponatinib have shown similar amino acid residues—namely THR94, LEU92, VAL35, and ASP160—via hydrogen bonding and van der Waals interactions with RIPK3 complex. These data demonstrate that both prunetin (activator) and ponatinib (inhibitor) bind to the allosteric site of the protein RIPK3 and execute their respective mechanism. This molecular docking analysis explains the mechanism of activation of RIPK3 as a preliminary in silico confirmation on its interaction with PRU.

**Figure 8:**
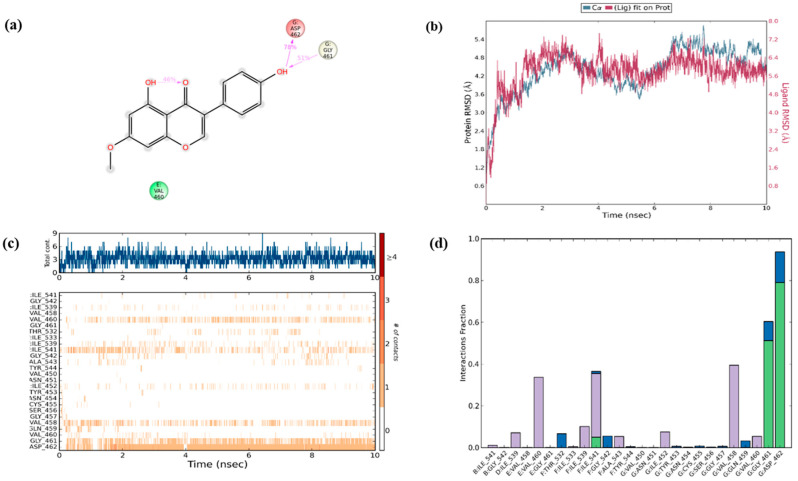
In silico molecular dynamics simulation. (**a**) Ligplot 2D summary showing the interaction of amino acids in the protein with the ligand. (**b**) The protein RMSD plot showing the ligand protein fit up to 10 ns during molecular dynamic simulation. (**c**) Protein–ligand interaction timeline with intensity of the each interacting amino acids during molecular dynamic simulation. (**d**) Interaction fractions of various residues during molecular dynamic simulation.

To secure the results obtained from molecular docking, the molecular dynamics simulations were performed on the ligand–receptor complex. The MD simulation was initiated on the bound complex and the behavior was monitored for a period of 10 ns. The stability of the complex is determined in terms of RMSD and RMSF of the protein along with the ligand. Root mean square fluctuation (RMSF) is useful for characterizing the local changes along the protein chain and root mean square deviation (RMSD) is used to measure the average change in displacement of selection of atoms for particular frame with respect to reference frame [47]. The protein ligand fit was observed to be in stable confirmation up to 10 ns and the stability was at a higher RMSD value as shown in Figure 8b. 

Over the simulation period, the protein–ligand contact ranges from one to more than nine contacts with the most interacted amino acid being ASP 462, followed by GLY 461 and VAL 458 as shown in Figure 8a. In addition, interaction with amino acids such as ILE 541, ILE 539, VAL 460, and ILE 452 had negligible impact on the overall interaction profile (Figure 8c). Furthermore, in the simulated model, the hydrogen bonds were recorded efficiently with GLY 461 and ASP462; however, only a transient impact was recorded on ILE 541 hydrophobic interactions were prominent with VAL 460, ILE 541, and VAL 458 amino acids. Water bridges were recorded with minor impact on ASP 462, GLY 461, THR 532, and GLY 542 (Figure 8d). During the period of 10 ns simulation, the deviations in the molecular properties—such as ligand RMSD, radius of gyration (rGyr), molecular surface area (MolSA), solvent accessible surface area (SASA), and polar surface area (PSA)—were recorded with minimal range with few intramolecular hydrogen bonds, provided in Figure S2. Collectively, the results of in silico validation through dynamics simulation of the docked complex supports the in vitro outcomes by confirming the effective interaction of PRU with RIPK3. 

With these impressive results observed by PRU, we also predicted its targets using Swiss Target Prediction (http://www.swisstargetprediction.ch/). Interestingly, the compound PRU has a high number of predicted class of targets in the kinase family of proteins which is shown in Figure S3. Structurally, the protein RIPK3 is a third member of RIPK kinase family of proteins comprised if N-terminal kinase domain with a unique C-terminal domain that differs from other protein domains [48]. This further adds significance to our study with our target RIPK3 that belongs to the kinase family of proteins [49]. 

Similarly, the pharmokinetic properties and drug-likeliness of the compound PRU was analyzed using SwissADME online version (http://www.swissadme.ch/) and the predicted data are shown in Figure S4. The bioavailability radar, as shown in Figure S4a, depicts the suitable physicochemical space for oral bioavailability of the compound PRU in colored zone including parameters such as flexibility, lipophilicity, saturation, size, polarity, and solubility. The solubility properties based on the consensus log *p* value of 2.43 in lipophilicity and log S (ESOL) value of −3.92, overall it can state that the compound PRU has a good lipophilic character and belongs to the water soluble class [50]. 

According to the pharmacokinetic properties represented in Figure S4b using the boiled egg model, the compound PRU shows high gastrointestinal absorption and it is slightly permeable to the blood–brain barrier. From Figure S4c, it can be seen that the compound PRU is not a P-gp substrate which means there would not be any issue in the excretion of the drug, since P-gp appears to have a significant role in limiting the cellular uptake of drugs from blood circulation and excretion [51]. Also, essential information about the interaction with cytochrome P450 isoforms (CYP1A2, CYP2C19, CYP2C9, CYP2D6, CYP3A4) has been predicted, because inhibition of these enzymes is certainly an important cause of drug interaction leading to toxicity [52]. The drug likeness parameter was found to be high as it follows the rule of five Lipinski, Ghose, Veber, Egan, and Mugge with a bioavailability score of 0.55. Based on the in silico ADMET analysis, it was found that the compound accomplished the ADMET descriptors criteria at an optimal level with no violations [53]. In combination, the results presented above demonstrate the anti-proliferative effect of compound PRU on AGS gastric cancer cells elucidated in vitro with supporting in silico validation.

## 4. Conclusions

In conclusion, the compound prunetin (PRU) was shown to be efficacious and executes anti-proliferative action on AGS cancer cells. PRU was found to induce necroptosis mediated cell death by the activation of RIPK3 protein, leading to the phosphorylation of MLKL which was confirmed in vitro and validated by in silico molecular docking and simulation studies. Furthermore, the cell death induced by PRU associated with the elevation of ROS generation through JNK activation was also confirmed. Additional analysis on the ADMET properties of PRU gives insight about the drug likeness of the compound. Thus, collectively, the data obtained from the study demonstrate a preliminary approach that PRU possesses anti-proliferative effect as and with further validations it would be potential in the treatment of gastric cancer.

## Supplementary Materials

The following are available online at https://www.mdpi.com/2218-273X/10/7/1086/s1, Figure S1: Cellular Morphological changes observed under light microscope upon three different concentrations of PRU (20, 40 and 80 μM) on AGS cells, Figure S2: Fluctuations of the ligand properties during MD simulation for 10ns, Figure S3: Swiss target prediction of compound Prunetin with its top class of predicted targets and Figure S4: ADME prediction of the compound PRU.
