# Supplementary material for: Compound Prunetin Induces Cell Death in Gastric Cancer Cell with Potent Anti-Proliferative Properties: In Vitro Assay, Molecular Docking, Dynamics, and ADMET Studies
Source: Biomolecules. 2020 Jul 21;10(7):1086. doi: 10.3390/biom10071086 (PMC7408406; doi:10.3390/biom10071086)
Supplement: Supplementary file 1 [file biomolecules-10-01086-s001.pdf]

biomolecules

Supplementary Materials

Compound Prunetin Induces Cell Death in Gastric Cancer Cell with Potent Anti-Proliferative Properties: In Vitro Assay, Molecular Docking, Dynamics, and ADMET Studies

Preethi Vetrivel [1], Seong Min Kim [1], Sang Eun Ha [1], Hun Hwan Kim [1], Pritam Bhagwan Bhosale [1], Kalaiselvi Senthil [2] and Gon Sup Kim [1,*]

[1] Research Institute of Life science and College of Veterinary Medicine, Gyeongsang National University, Gazwa, Jinju 52828, Korea

[2] Avinashilingam Institute for Home Science and Higher Education for Women, Department of Biochemistry, Biotechnology and Bioinformatics, Coimbatore 641043, India

* Correspondence: gonskim@gnu.ac.kr; Tel.: 010-3834-5823

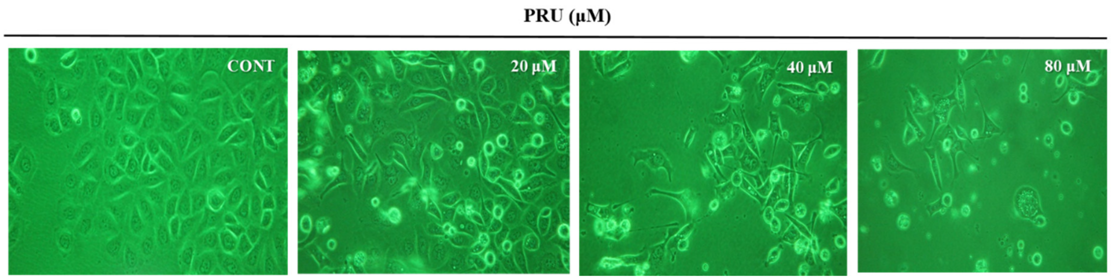

Figure S1. Cellular Morphological changes observed under light microscope upon three different concentrations of PRU (20, 40 and 80 μM) on AGS cells.

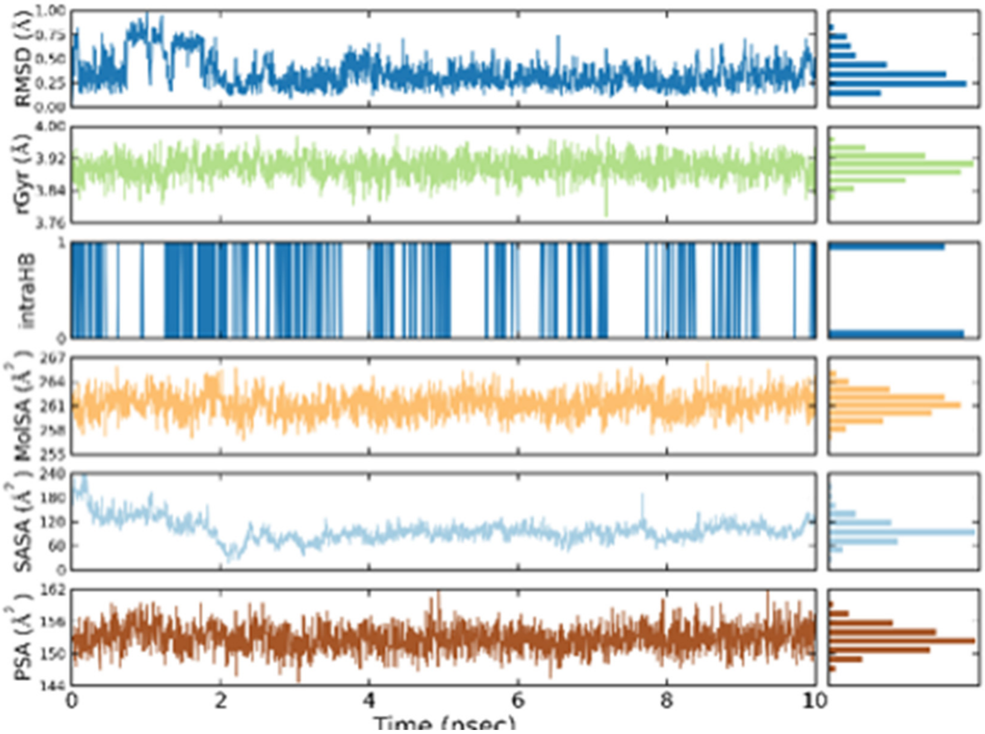

Figure S2. Fluctuations of the ligand properties during MD simulation for 10ns.

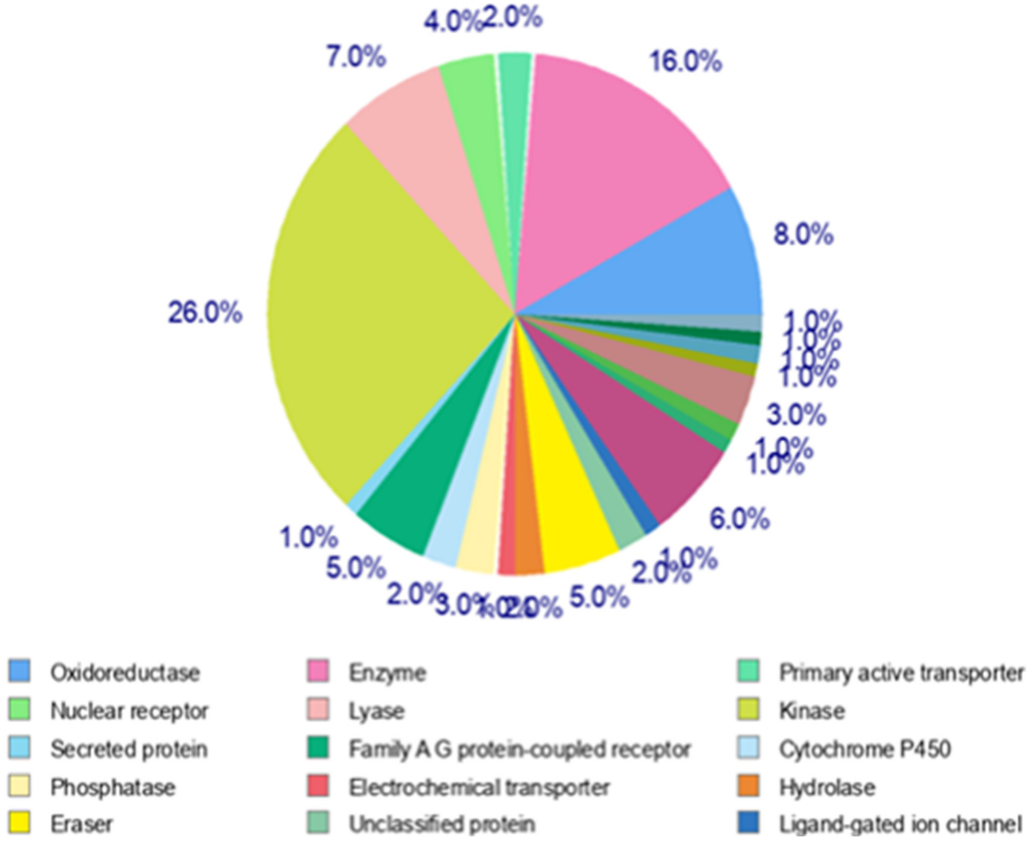

Figure S3. Swiss target prediction of compound Prunetin with its top class of predicted targets.

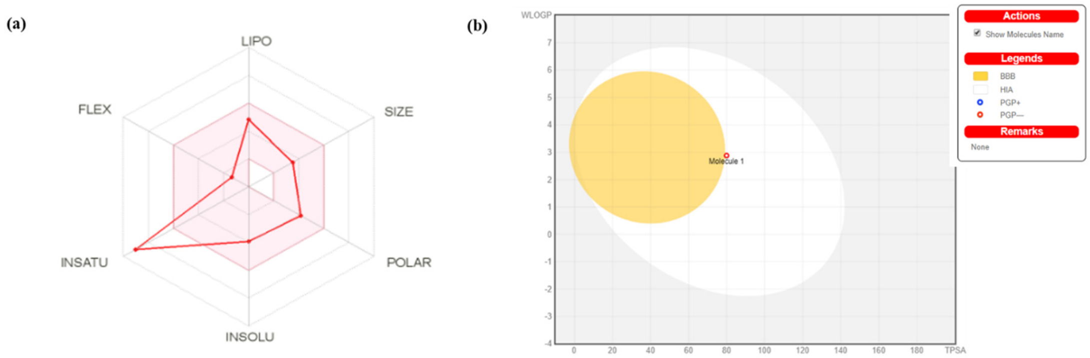

(c)

	Pharmokinetics								
	**GI absorption**	**BBB permeant**	**p-gp**	**CYP1A2 inhibitor**	**CYP2C19 inhibitor**	**CYP2C9 inhibitor**	**CYP2D6 inhibitor**	**CYP3A4 inhibitor**	**Log Kp (skin permeation)**
**Prunetin**	High	No	No	Yes	No	No	Yes	Yes	-5.91 cm/s

	Drug-likeness & Medicinal Chemistry									
	**Lipinski**	**Ghose**	**Veber**	**Egan**	**Muegge**	**Bioavailability score**	**PAINS**	**Brenk**	**Leadlikeness**	**Synthetic accessibility**
**Prunetin**	Yes	Yes	Yes	Yes	Yes	0.55	0 alert	0 alert	Yes	2.96

Figure S4. ADME prediction of the compound PRU.

© 2020 by the authors. Licensee MDPI, Basel, Switzerland. This article is an open access article distributed under the terms and conditions of the Creative Commons Attribution (CC BY) license (http://creativecommons.org/licenses/by/4.0/).
